# Supplementary material for: Imbalance in the response of pre- and post-synaptic components to amyloidopathy
Source: Sci Rep. 2019 Oct 16;9:14837. doi: 10.1038/s41598-019-50781-1 (PMC6795896; doi:10.1038/s41598-019-50781-1)

**Imbalance in the response of pre- and post-synaptic components to amyloidopathy**

Terri-Leigh Stephen^1,4#^, Francesco Tamagnini^1,2,3#^, Judith Piegsa^1,3^, Katherine Sung^1^, Joshua Harvey^1^, Alice Oliver-Evans^1^, Tracey K. Murray^1^, Zeshan Ahmed^1^, Michael L. Hutton^1^, Andrew Randall^3^, Michael J. O’Neill^1,5^, Johanna S. Jackson^1,6^*

^1^Eli Lilly and Company, Lilly Research Centre, Erl Wood Manor, Windlesham, Surrey, GU20 6PH, UK

^2^University of Reading, School of Pharmacy, Whiteknights Campus, Hopkins Building, Reading RG6 6LA, UK

^3^Institute of Biomedical and Clinical Sciences, University of Exeter Medical School, Hatherly Laboratories, University of Exeter, Exeter EX4 4PS, UK.

^4^Current address: Department of Gerontology, University of Southern California, Los Angeles, California 90089, USA

^5^Current address: AbbVie Deutschland GmbH & Co. K.G., Ludwigshafen, Germany

^6^Current address: UK Dementia Research Institute at Imperial College, Department of Brain Sciences, Imperial College London, London, UK

^#^co-first author

*Corresponding author: johanna.jackson@imperial.ac.uk

**Imbalance in the response of pre- and post-synaptic components to amyloidopathy**

**Supplementary Figure 1**

Surface area of individual amyloid plaques in J20 mice in the early (a) and late (b) groups. P values calculated using paired t-tests (these values have not been corrected for multiple comparisons as they are 2 orders of magnitude below the threshold of 0.05).

**SI Table 1. Pairwise comparisons between age-groups and plaque-initial-size groups.** Top. In younger animals, small plaques grew significantly faster, in comparison to large ones and almost reached significance in comparison to medium ones. No difference was however observed between medium and large-sized plaques. Bottom. No effect of plaque’s initial size on growth rate was observed at a later age-stage.

**SI Table 2. Pairwise comparisons between age-groups and plaque-sectors.** Top. The core of the plaque does not grow at different rates between younger and older animals; however, the cloud sector grows faster in younger animals. Bottom. Both in younger and older animals, the cloud sector grows faster than the core.

**Supplementary Figure 1**


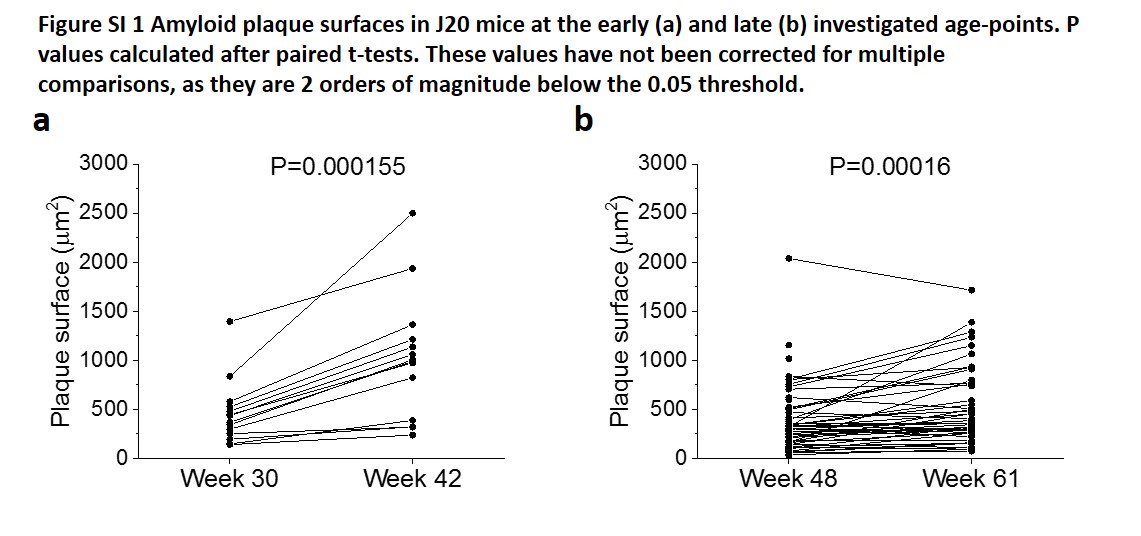


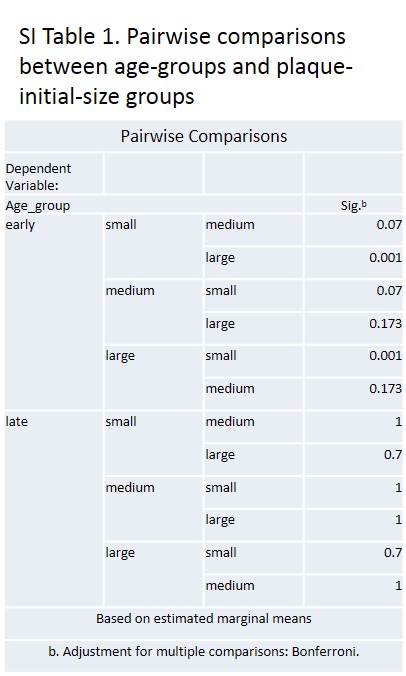

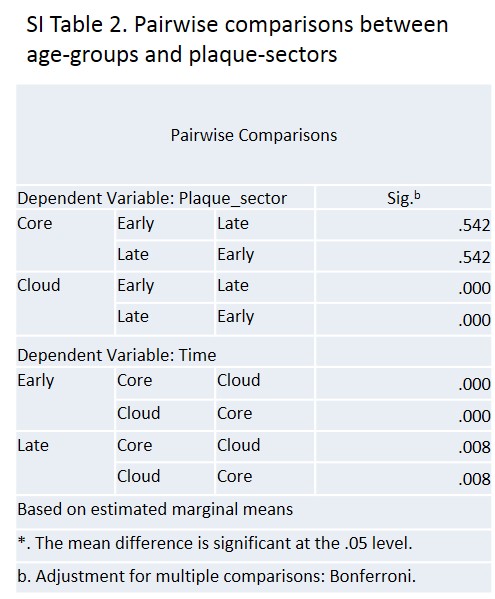

Supplement: Supplementary file 1 — Supplementary Figure 1 [file 41598_2019_50781_MOESM1_ESM.docx]
